# *Actinidia callosa* var. *callosa* suppresses metastatic potential of human hepatoma cell SK-Hep1 by inhibiting matrix metalloproteinase-2 through PI3K/Akt and MAPK signaling pathways

**DOI:** 10.1186/s40529-017-0216-4

**Published:** 2018-01-22

**Authors:** Jeng-Shyan Deng, Jui-Shu Chang, Jung-Chun Liao, Wei Chao, Ming-Ming Lee, Chien-Hua Cheng, Guan-Jhong Huang

**Affiliations:** 1 Department of Food Nutrition and Health Biotechnology, Asia University, Taichung, Taiwan; 2 School of Chinese Medicine, Graduate Institute of Integrated Medicine College of Chinese Medicine, China Medical University, Taichung, Taiwan; 3 School of Pharmacy, College of Pharmacy, China Medical University, Taichung, Taiwan; 4 School of Chinese Pharmaceutical Sciences and Chinese Medicine Resources, College of Chinese Medicine, China Medical University, Taichung, Taiwan; 5 Department of Visual Communication Design, Asia University, Taichung, Taiwan

**Keywords:** *Actinidia callosa* var. *callosa*, Migration, Invasion

## Abstract

**Background:**

Cancer cell metastasis involving multi-step procedures and cytophysiological property changes may make difficult in the clinical management and death rate increasing.

**Results:**

In this study, we first observed that ethyl acetate fraction of *Actinidia callosa* var. *callosa* (EAAC) carry out a dose-dependent inhibitory effect without cytotoxicity on the mobility and invasion of highly metastatic SK-Hep1 cells. To investigate the EAAC in cancer metastasis, SK-Hep1 cells were treated with EAAC at various concentrations and then subjected to gelatin zymography, casein zymography and western blot to study the impacts of EAAC on metalloproteinase-2 (MMP-2) and tissue inhibitor of metalloproteinase-1/2 (TIMP-1/2), respectively. Our results showed that EAAC treatment may decrease the expressions of MMP-2 and enhance the expression of TIMP-1/2 in a concentration-dependent manner. EAAC also inhibited effect on the phosphorylation of mitogen-activated protein kinase (MAPK) and phosphatidylinositol-3-kinase/serine/threonine protein kinase [or protein kinase B (PI3K/Akt)] and focal adhesion kinase (FAK).

**Conclusions:**

These results indicate that EAAC inhibited SK-Hep1 cell of metastasis by reduced protein level of MMP-2 through the suppression of MAPK and FAK signaling pathway and of the activity of PI3K/Akt. These findings suggest that EAAC may be used as an antimetastatic agent.

## Background

Hepatocellular carcinoma (HCC) is the most common type of cancer in the Southeast Asia, including Taiwan. Metastasis is a characteristic of highly malignant cancers associated with a poor clinical outcome. Malignant tumor progression relies on the capacity of cancer cells to invade and promoting angiogenic effects in tumor areas. One characteristic that metastatic cancer cells were induced the dissolution of basement membrane proteins and the extracellular matrix (ECM). The activity of the degradative process is mediated by matrix metalloproteinases (MMPs) (Forrbes et al. 2003), which belong to zinc-dependent neutral endopeptidases that can break down the ECM (Parmo-Cabanas et al. 2006). The MMPs are found as mediators of tumor migration and invasion by breaking down connective tissue barriers (Chao et al. 2017). Thus, the inhibition of MMP activity is important for the prevention of cancer metastasis. MMP-2 protein expressions are high level in hepatoma cells, such as SK-Hep1 cells (Ho et al. 2012). In addition, urokinase-type plasminogen activator (uPA) is a serine protease that activation of pro-uPA occurs after binding to its receptor uPAR (uPA receptor). Plasminogen activator inhibitors (PAI-1 and PAI-2) were important component of the coagulation system that inhibits both receptor-bound and free uPA (Liao et al. 2009). Meanwhile, Tissue inhibitors of metalloproteinases (TIMPs) control MMP activities and the imbalance between MMPs and TIMPS may contribute to degradation or depositions of ECM involved in tissue remodeling and regulate tumor cell progression including tumor angiogenesis (Shih et al. 2009).

*Actinidia callosa* var. *callosa* (*Actinidiaceae*; AC) is a liana plant, which belongs to Actinidiaceae, the roots of AC have been used in traditional Chinese medicine for the treatment of anti-pyretic, anti-inflammatory diseases and various cancers, including gastric carcinoma, liver carcinoma, and breast carcinoma (Liao et al. 2013; Lee et al. 2014). Actinidia species had showed some pharmacological effects such as anti-inflammatory activity from the fruit of *Actinidia polygama* (Ren et al. 2007), antitumor, and immunomodulatory activity from the roots of *Actinidia eriantha* (Xu et al. 2009). In the present study, we used ethyl acetate fraction of AC (EAAC) to investigate the antimetastatic effects on a human hepatocarcinoma SK-Hep1 cell line in vitro.

## Methods

### Chemicals

Trypsin, EDTA, fetal bovine serum (FBS) and penicillin/streptomycin were from Gibco Life Technologies, Inc. (Paisley, UK). Cell culture supplies were purchased from Costar (Corning, Inc., Cypress, CA, USA). Dulbecco’s modified Eagle’s medium (DMEM), 3-(4,5-dimethylthiazolyl-2)-2,5-diphenyltetrazolium bromide (MTT), RNase A, and other chemicals were obtained from Sigma Chemical Co. (St. Louis, MO, USA). Plant materials were collected from Taichung Country in Taiwan. They were identified and authenticated by Dr. Shyh-Shyun Huang, Professor, School of Pharmacy, College of Pharmacy, China Medical University, Taichung, Taiwan.

### Extraction and fractionation

The powder of *A. callosa* var. *callosa* (AC) (2 kg) was extracted with methanol (MAC) three times. The extract was evaporated under reduced pressure using a rotavapor, and then stored under light protection. A yield equivalent to 6.51% of the original weight was obtained. Next, MAC (130.2 g) was dissolved and suspended in 100 mL of water in a separatory funnel prior to being partitioned in sequence with *n*-hexane, ethyl acetate and *n*-butanol (800 mL each for three times). Under reduced pressure, fractions were yielded and collected: *n*-hexane fraction (17.79 g, 13.66%), ethyl acetate fraction (78.50 g, 60.73%), *n*-butanol fraction (17.18 g, 13.21%) and aqueous fraction (16.73 g, 12.76%). All extracts were stored in the refrigerator before the use.

### Fingerprint analysis by HPLC

The analysis will be performed on a HITACHI HPLC L-5000 system equipped with a degasser, pumps, and a photodiode array detector linked to a PC computer running the software program HPLC LACHROM. The analytical column (250 × 4.6 mm i.d.) used is Mightysil 5 μm C18 (Japan). For HPLC analysis, an aliquot (10 μL) is injected into the columns and eluted at 30 °C. Mobile phase eluent (A) is 0.6‰ aqueous phosphoric acid, and eluent (B) MeOH, and the flow rate is kept constant throughout the analysis at 1 mL/min. Injections are accomplished with a 10 μL fixed loop. The elution programme used is as follows 13% A to 87% B. For photodiode array detection, the wavelengths of triterpenic acids at their respective maximum absorbance–wavelength can monitored at the same time. Identification is based on retention times and on-line spectral data in comparison with authentic standards. Quantification is performed by establishing calibration curves for each compound determined, using the standards.

### Cell culture

The hepatocarcinoma SK-Hep1 cell was purchased from the Bioresources Collection and Research Center (BCRC) of the Food Industry Research and Development Institute (Hsinchu, Taiwan). Cells were cultured in plastic dishes containing DMEM supplemented with 10% fetal bovine serum (FBS) in a CO_2_ incubator (5% CO_2_ in air) at 37 °C and subcultured every 2 days at a dilution of 1:5 using 0.05% trypsin–0.02% EDTA in Ca^2+^-, Mg^2+^-free phosphate-buffered saline (DPBS).

### Cell viability

The cells (2 × 10^5^) were cultured in 96-well plate containing DMEM supplemented with 10% FBS for 1 day to become nearly confluent. Then cells were cultured with EAAC for 24 h. After that, the cells were washed twice with DPBS and incubated with 100 µL of 0.5 mg/mL MTT for 2 h at 37 °C testing for cell viability. The medium was then discarded and 100 µL dimethylsulfoxide (DMSO) was added. After 30-min incubation, absorbance at 570 nm was read by a microplate reader.

### Determination of MMP by zymography

MMP released from SK-Hep1 cells into the medium was assayed by gelatin zymography (7.5% zymogram gelatin gels) (Hung et al. 2009). Briefly, the culture medium was electrophoresed in a 10% SDS-PAGE gel containing 0.1% gelatin. The gel was then washed at room temperature in a solution containing 2.5% (*v/v*) Triton X-100 with two changes and subsequently transferred to a reaction buffer for enzymatic reaction containing 1% NaN_3_, 10 mM CaCl_2_ and 40 mM Tris–HCl, pH 8.0, at 37 °C with shaking overnight (for 12–15 h). Finally, the MMP gel was stained for 30 min with 0.25% (*w/v*) Coomassie blue in 10% acetic acid (*v/v*) and 20% methanol (*v/v*) and destained in 10% acetic acid (*v/v*) and 20% methanol (*v/v*).

### Cell migration assay

Tumor cell migration was assayed in transwell chambers (Millipore) (Hung et al. 2009). Transwell chambers with 6.5 mm PVPP-free polycarbonate filters of 8 µm pore size were used. SK-Hep1 cells (5 × 10^5^/mL) and 0–200 µg/mL of EAAC were suspended in DMEM (100 µL, serum free), placed in the upper transwell chamber, and incubated for 24 h at 37 °C. Then, the cells on the upper surface of the filter were completely wiped away with a cotton swab, and the lower surface of the filter was fixed in methanol, stained with Giemsa, and counted under a microscope at a magnification of 200×. For each replicate, the tumor cells in 10 randomly selected fields were determined, and the counts were averaged.

### Cell invasion assay

The invasion of tumor cells was assessed in transwell chambers containing a 6.5 mm PVPP-free polycarbonate filter with 8 µm pore size, as described in the cell migration assay (Ho et al. 2012) except that each filter was coated with 100 µL of a 1:20 diluted Matrigel in cold DMEM to form a thin continuous film on top of the filter. The number of cells was adjusted to 5 × 10^5^/mL and 100 µL (containing 5 × 10^4^ cells) was transferred to each of triplicate wells in DMEM containing 10% FBS. After incubating for 24 h, the cells were stained and counted as described above, and the number of cells invading the lower side of the filter was measured.

### Preparation of whole-cell lysates

SK-Hep1 cells (1 × 10^5^ cells) were plated in a 100 mL tissue culture flask and were treated with various concentrations of EAAC and 0.2% DMSO. SK-Hep1 cells were washed twice with PBS and were scraped into a microcentrifuge tube. The cells were centrifuged at 1250*g* for 5 min, and the pellet was lysed with iced-cold RIPA buffer (1% NP-40, 50 mM Tris-base, 0.1% SDS, 0.5% deoxycholic acid, 150 mM NaCl, pH 7.5), to which was added freshly prepared phenylmethylsulfonyl fluoride (10 mg/mL), leupeptin (17 mg/mL), and sodium orthovanadate (10 mg/mL). After incubation for 5 min on ice, the samples were centrifuged at 10,000*g* for 10 min, and then the supernatants were collected as whole-cell lysates. The lysates were denatured and subjected to SDS-PAGE and western blotting. The protein content was determined with Bio-Rad protein assay reagent using BSA as a standard.

### Western blotting analysis

Whole-cell lysate proteins were mixed with an equal volume of electrophoresis sample buffer, and the mixture was then boiled for 10 min. Then, an equal protein content of total cell lysate from control, 0.2% DMSO, and EAAC-treated sample were resolved on 10–12% SDS-PAGE gels. Proteins were then transferred onto nitrocellulose membranes (Millipore, Bedford, MA) by electroblotting using an electroblotting apparatus (Bio-Rad). Nonspecific binding of the membranes was blocked with Tris-buffered saline (TBS) containing 1% (*w/v*) nonfat dry milk and 0.1% (*v/v*) Tween-20 (TBST) for more than 2 h. Membranes were washed with TBST three times each for 10 min and then incubated with an appropriate dilution of specific primary antibodies in TBST overnight at 4 °C. The membranes were washed with TBST and then incubated with an appropriate secondary antibody (horseradish peroxidase-conjugated, goat antimouse, or antirabbit IgG) for 1 h. After washing the membrane three times each for 10 min in TBST, the bands were visualized using ECL reagents (Millipore, Billerica, MA). Band intensity on scanned films was quantified using Kodak molecular imaging (MI) software and expressed as relative intensity compared with control.

### Statistical analysis

Experimental results were presented as the mean ± standard deviation (SD) of three parallel measurements. A difference was considered to be statistically significant when *p* < 0.05, *p* < 0.01, and *p* < 0.001.

## Results

### Compositional analysis of EAAC by HPLC

**Fig. 1:**
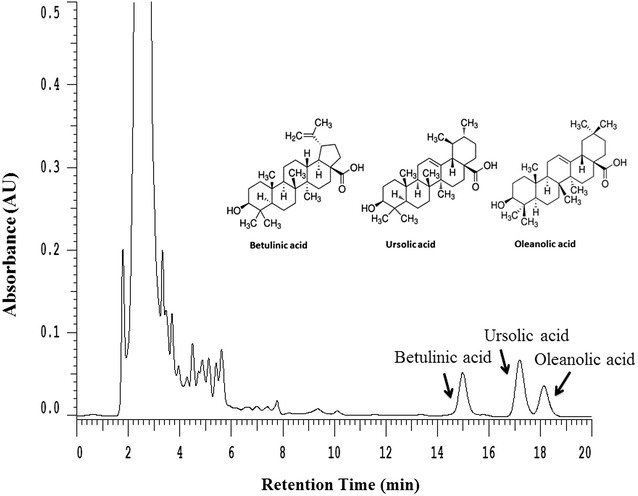
HPLC chromatograms (280 nm) of ethyl-acetate fraction of the stem of *A. callosa* var. *callosa* (EAAC)

In this study, we set up the fingerprint chromatogram in the EAAC. The three compounds of betulinic acid, ursolic acid, and oleanolic acid were used as markers. As shown in Fig. 1, these triperpenic acids have been identified as betulinic acid (retention time, 15.16 min), ursolic acid (17.43 min), and oleanolic acid (18.42 min) by their retention time and UV absorbance of purified standards. According to the plot of peak–area ratio (*y*) vs. concentration (*x*, µg/mL), the regression equations of the three constituents and their correlation coefficients (*r*) were determined. The relative amounts of the three triperpenic acids found in EAAC was in the order of ursolic acid (6.34 mg/g) > oleanolic acid (5.00 mg/g) > betulinic acid (1.25 mg/g), respectively.

### Effect of EAAC on cell proliferation

**Fig. 2:**
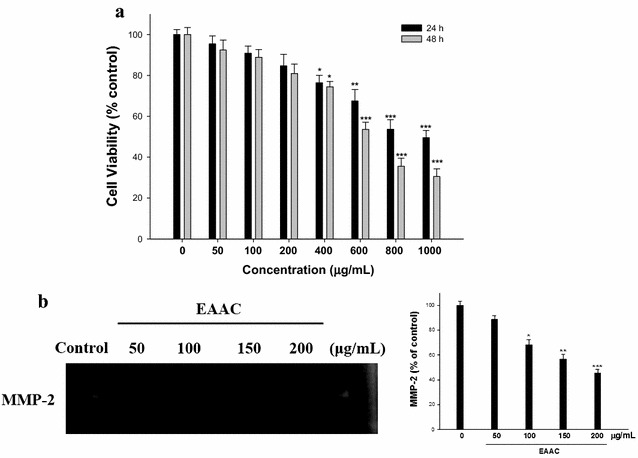
Effects of EAAC on SK-Hep1 cell viability (**a**) and MMP-2 (**b**) activity. The cells were incubated for 24 and 48 h in the absence or present of EAAC (0, 50, 100, 150, and 200 μg/mL). After treatment, cell viability assay was performed using MTT assay. Optical density was determined at 570 nm and is expressed as cell survival relative to control. Cells were treated with various concentrations of EAAC for 24 h. The conditioned media were collected, and MMP-2 activity was determined by gelatin zymography. MMP-2 activity was quantified by densitomeric analysis. The data were presented as mean ± SD for three different experiments performed in triplicate. *p < 0.05, **p < 0.01, and ***p < 0.001 was compared with untreated control group

In this study, the cytotoxicity of EAAC toward SK-Hep1 human hepatoma cells was evaluated using the MTT assay. The effects of 0–1000 µg/mL EAAC on cell growth after 24 and 48 h are shown in Fig. 2a. After 24 and 48 h of incubation, EAAC inhibited cell proliferation in a dose-dependent fashion, with cell numbers significantly reduced by 4.6–50.5 and 7.5–69.4% compared to the control. EAAC has been shown to have anti-tumor effects in vitro, but the underlying mechanism is unclear. In this study, we demonstrated that EAAC inhibited the proliferation of SK-Hep1 cells in a concentration-dependent manner. It was therefore clear that a 24 h treatment of EAAC, at a concentration ranging from 0 to 200 µg/mL, has no cytotoxicity to SK-Hep1 cells, highly metastatic cancer cells.

### EAAC inhibits the activation of MMP in SK-Hep1 cells

To check the possible antimetastatic mechanisms of EAAC, we determined the effect of these compounds on the expression of MMP. First, cultured conditioned media of SK-Hep1 cells were subjected to zymographic analysis. After 24 h, treatment with 0–200 µg/mL EAAC shows a dose–response relationship in MMP-2 (Fig. 2b). Treatment of EAAC at 50, 100, 150, and 200 µg/mL decreased MMP-2 expression by 11.5, 31.6, 44.5 and 54.5%, respectively. Thus, in the SK-Hep1 cell system, MMP-2 was down-regulated by EAAC treatments. In addition, we did not find MMP-9 activity in the zymography assay.

### Effect of EAAC on migration and invasion of SK-Hep1 cells in vitro

**Fig. 3:**
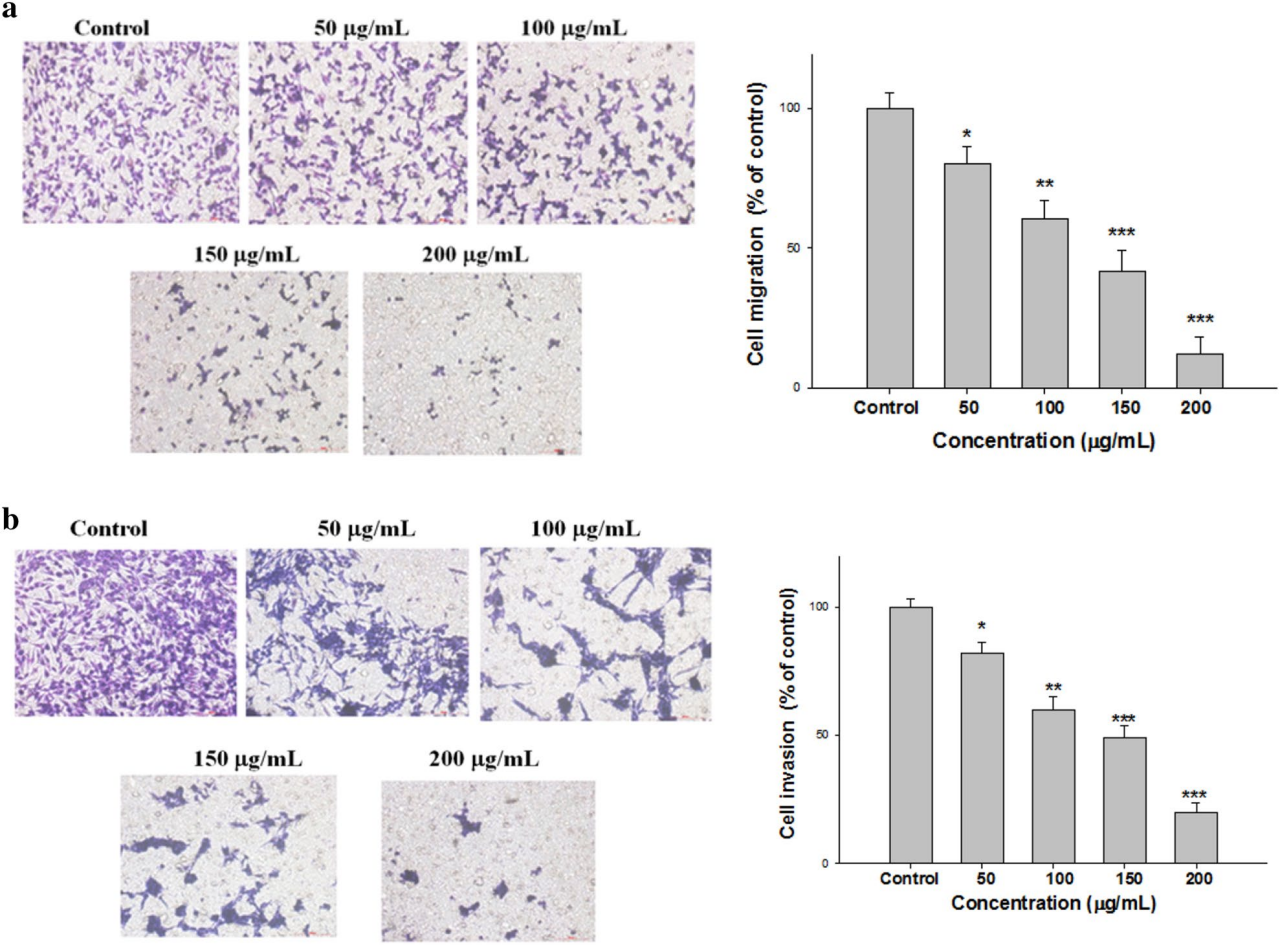
Effects of EAAC on transwell migration (**a**) and invasion (**b**) of SK-Hep1 cells. SK-hep1 cells were incubated with EAAC (0, 50, 100, 150, and 200 μg/mL) for 24 h, and the transwell migration cells and invasion cells were counted. The data were presented as mean ± SD for three different experiments performed in triplicate. *p < 0.05, **p < 0.01, and ***p < 0.001 were compared with untreated control group

The transwell assay was used to study the migration and invasion of SK-Hep1 cells 24 h after EAAC treatment. We found that EAAC at 0–200 µg/mL significantly decreased both the migration and invasion (Fig. 3a, b) of SK-Hep1 cells in dose-dependent manners. The IC_50_ values for EAAC on migration and invasion of SK-Hep1 was approximately 73.3 and 92.7 µg/mL, respectively.

### Effect of EAAC on MMPs, TIMPs, and FAK expression in SK-Hep1 cells

**Fig. 4:**
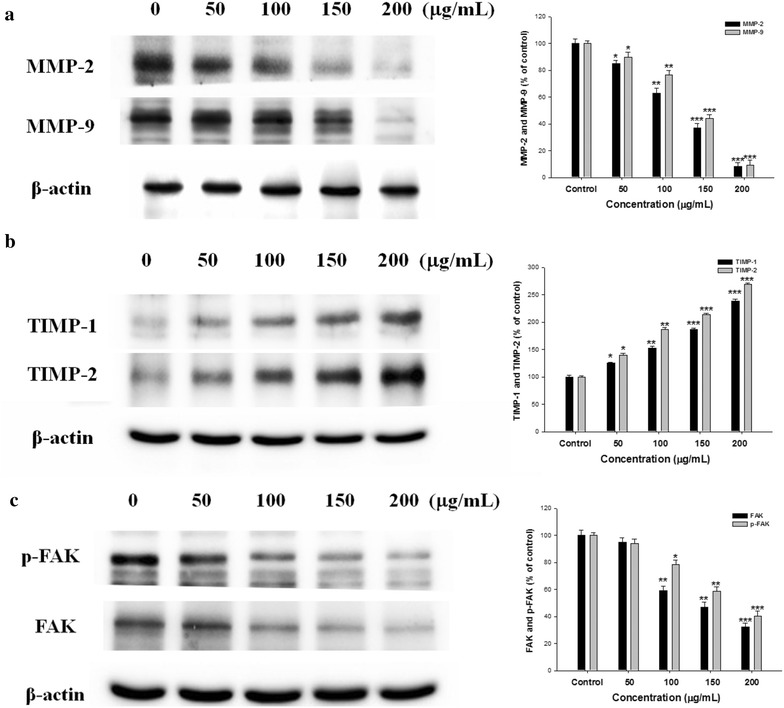
Effects of EAAC on MMP (**a**), TIMP (**b**), and FAK (**c**) protein expressions. Analyzed by western blot, SK-Hep1 cells were treated with 0, 50, 100, 150, and 200 μg/mL for 24 h and cell lysates were subjected to SDS-PAGE followed by western blotting. Activities of these proteins were subsequently quantified by densitometric analyses, with the control set to 100%. The data were presented as mean ± SD for three different experiments performed in triplicate. *p < 0.05, **p < 0.01 and ***p < 0.001 0.001 were compared with untreated control group

To further search the modulation of pro-MMP activation mediated by EAAC, we determined MMP-2/-9 and TIMP-1/-2 protein expression levels. A variety of scientific papers have revealed that over-expression of TIMPs, especially TIMP-1 and TIMP-2, can inhibit tumor growth, invasion and metastasis. As results shown in Fig. 4a, EAAC especially decreased MMP-2 and MMP-9 activity in a concentration-dependent manner while activity was also increased TIMP-1 and TIMP-2 by SK-Hep1 (Fig. 4b).

Focal adhesion kinase (FAK) is a potentially important therapeutic target because it is a key point of convergence for the relevant growth factor pathways required for survival and metastatic functions of cancer cells. FAK was recognized as a protein tyrosine kinase that place to cell focal adhesion contacts and adhesion sites (Shi et al. 2017). To evaluate the effect of EAAC on FAK protein expression, SK-Hep1 was treated with EAAC at 0–200 μg/mL for 24 h. EAAC suppressed FAK expression (Fig. 4c). We also illustrated this suppressive effect on the phosphorylation of FAK by EAAC in SK-Hep1 cell.

### Inhibition by EAAC of MAPK phosphorylation

**Fig. 5:**
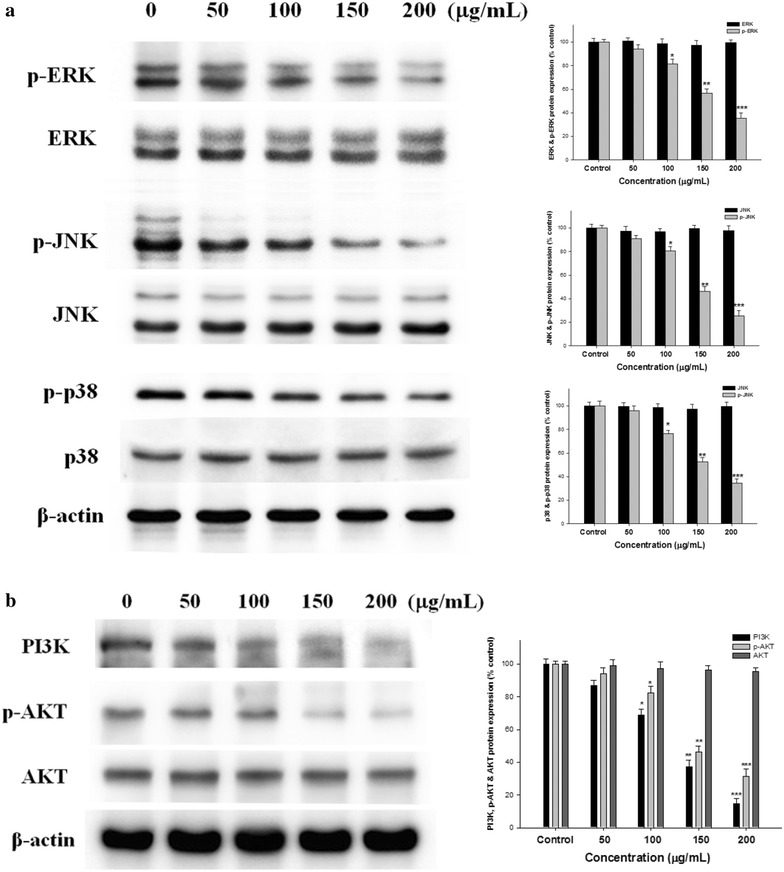
Inhibitory effect of EAAC on MAPK (**a**), and PI3K, AKT (**b**) signaling pathway. SK-Hep1 cells were cultured in serum-free media containing 0, 50, 100, 150, and 200 mg/mL EAAC for 24 h, and the cell lysates were subjected to SDS-PAGE followed by western blotting. Activities of these proteins were subsequently quantified by densitometric analyses, with the control set to 100%. The data were presented as mean ± SD for three different experiments performed in triplicate. *p < 0.05, **p < 0.01 and ***p < 0.001 were compared with untreated control group

To evaluate signaling pathway induced by EAAC in SK-Hep1 cells, we investigated the responses of MAPKs, which are signaling molecules receptive to extracellular stimuli. We dissected the phosphorylation of MAPKs in SK-Hep1 cells after treatment with EAAC (0–200 μg/mL) for 24 h. As shown in Fig. 5a, EAAC inhibited the phosphorylation activities of MAPKs in SK-Hep1 cells. Treatment with 200 μg/mL EAAC reduced the phosphorylation activity of ERK1/2, p38 and JNK1/2 about 64.5, 65.4 and 74.5%, respectively.

### Effects of EAAC on the PI3K/Akt signaling in SK-Hep1 cells

PI3K/Akt signaling pathway plays a role in facilitating tumor cell invasion and metastasis. The constitutive activation of PI3K/Akt signaling is frequently found in cancer (Wang et al. 2016). Incubation of SK-Hep1 cells with EAAC (200 µg/mL) decreased the PI3K and the phosphorylation of Akt protein levels (Fig. 5b). EAAC (200 µg/mL) produced suppression on PI3K and the phosphorylated of Akt level about 85.5 and 68.4% compared to control intensity at 24 h. EAAC inhibited the expression of PI3K and the phosphorylated of Akt was coincided with the kinetics of cell migration and invasion.

### Effect of betulinic acid, ursolic acid and oleanolic acid on MMP expression

**Fig. 6:**
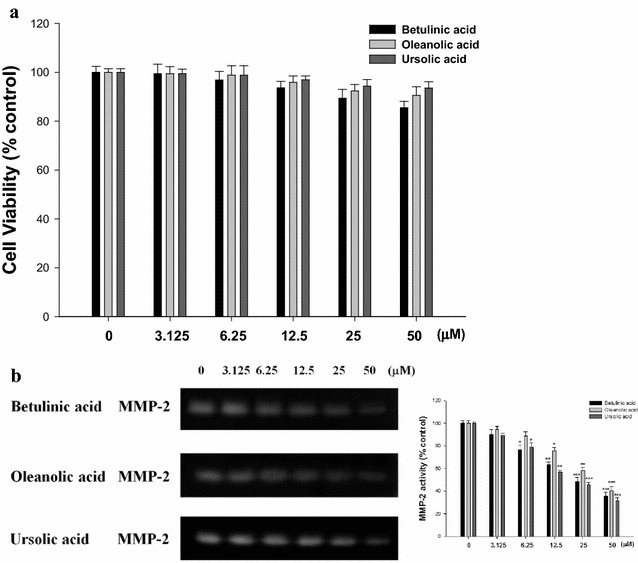
Effects of betulinic acid, ursolic acid, and oleanolic acid on SK-Hep1 cell MMP-2 activity. The cells were incubated for 24 h in the absence or present of betulinic acid, ursolic acid and oleanolic acid (0, 3.125, 6.25, 12.5, 25, and 50 μM). After treatment, cell viability assay was performed using MTT assay. Optical density was determined at 570 nm and is expressed as cell survival relative to control. The conditioned media were collected, and MMP-2 activity was determined by gelatin zymography. MMP-2 activity was quantified by densitomeric analysis. The data were presented as mean ± SD for three different experiments performed in triplicate. *p < 0.05, **p < 0.01, and ***p < 0.001 was compared with untreated control group

To investigate the possible anti-metastatic mechanisms of EAAC, we determined the effect of three compounds (betulinic acid, ursolic acid and oleanolic acid) on the activities of MMP-2. It was clear that a 24 h treatment of betulinic acid, ursolic acid and oleanolic acid, at a concentration ranging from 0 to 50 µM, has no cytotoxicity to SK-Hep1 cells (Fig. 6a). In addition, cultured conditioned media of SK-Hep1 cells were subjected to zymographic analysis. Treatment with 50 µM betulinic acid, ursolic acid and oleanolic acid after 24 h suppressed MMP-2 activity by 64.4, 59.4, and 68.4%, respectively. The value of half-maximal effective inhibitory concentration (IC_50_) for MMP-2 by betulinic acid, ursolic acid and oleanolic acid was approximately 23.5, 45.6, and 19.3 µM (Fig. 6b). Thus, in the SK-Hep1 cell system, MMP-2 was down-regulated by betulinic acid, ursolic acid and oleanolic acid treatments.

## Discussion

SK-Hep1 is an immortal, human cell line derived from the ascetic fluid of a patient with liver adenocarcinoma. High metastatic capacities of SK-Hep1 cells are suitable for cancer metastasis research and have been used wildly in the literature (Park et al. 2015). In this study we examined the antimetastatic effects of EAAC in human hepatoma SK-Hep1 cells. We found that EAAC inhibited the cell migration and cell invasion of SK-Hep1 cells without cytotoxicity by targeting the MAPK and PI3K/AKT signaling pathway. We showed that EAAC notably inhibited the activities of MMP-2. These results revealed that the antimetastatic effect of EAAC was associated with the inhibition of enzymatically degradative processes (MMP-2 and -9) of tumor metastasis. To our knowledge, this is the first study to demonstrate the biochemical mechanisms by which EAAC reduces the metastasis in SK-Hep1 cells.

MMP-2 and -9 are type IV collagenase/gelatinase, and their expression is associated with aggressive tumor phenotypes and poor prognosis (Chen et al. 2015). These enzymes are markers associated with the tumor invasion and metastasis and degrade the environmental ECM and the basement membrane. MMP-2 activity is the highest expressions in the tissue of hepatocellular carcinoma with metastasis and plays a key role in the degradation of the basement membrane and ECM, thereby enhancing migration of endothelial cells (Byambaragchaa et al. 2013). Our results showed that EAAC at 0–200 µg/mL significantly inhibited MMP-2 activity of SK-Hep1 cells, significantly. Thus, it is likely that inhibition of MMP-2 activities by EAAC is responsible for EAAC inhibition of migration and invasion of the SK-Hep1 cells.

FAK is involved in ECM via expression and release of MMP-2 and MMP-9 and suggested to have an essential role in metastasis (Huang et al. 2010). Activated FAK binds to PI3K domain 2 (SH2) where it catalyzes the phosphorylation of inositol lipids (Casar et al. 2014). Thus, FAK also regulates phosphorylation of ERK and Akt, and the FAK-ERK and FAK-Akt signaling pathway induces MMP-9 expression (Lu et al. 2016). In this study, we found that EAAC inhibited the activation of FAK, as evidenced by reduced phosphorylation of FAK. We also demonstrated that treatment with EAAC inhibited phosphorylation of ERK1/2, JNK1/2, and p38 activity. The results indicated that EAAC inhibited the phosphorylation of ERK1/2, p38, and JNK expressions. MAPK kinases associated with MMP-2 and MMP-9 expression in protein levels.

PI3K/Akt signaling pathways is the chief mechanism for controlling cell survival, metabolism, angiogenesis and malignant transformation in response to extracellular cues (Hseu et al. 2017). Akt is a protein kinase, as an important target gene of PI3K, which phosphorylates phosphatidylinositol-4, 5-bisphosphate (PIP2) to form phosphatidylinositol 3, 4, 5-trisphosphate (PIP3) (He et al. 2017). Dysregulation of the PI3K/Akt pathway is implicated in a number of human diseases including cancer, diabetes, cardiovascular disease and neurological diseases. Thus, we evidenced that FAK promotes SK-Hep1 cancer cell migration and invasion in concert with the activation of the MAPK and PI3K/Akt signaling pathways, which modulate the MMP expression. In addition, we showed that EAAC inhibited PI3K/Akt in SK-Hep1 cells.

Plants provide a vigorous source for food health and drug discovery. One important class of bioactive phytochemicals is triterpenoids, which represent a large family of compounds classified according to the number of isoprene units (Chen et al. 2016). Triterpenoids are widely spread out and 20,000 triterpenoids have been identified from the various parts of medicinal plants (Gopalakrishnan and Thomas 2014). In this study, three triperpenic acids (ursolic acid, oleanolic acid and betulinic acid) found in EAAC and there biosynthesized by the acetate/mevalonate pathway and (3*S*)-2,3-oxidosqualene cyclization. Ursolic acid, oleanolic acid and betulinic acid possess interesting pharmacological properties, including the antioxidant, microbicide, antidiabetic, anti-inflammatory, hypolipidemic, anticancer and antiatherosclerotic actions (Silva et al. 2016).

Betulinic acid, ursolic acid and oleanolic acid are pentacyclic triterpene acids naturally occurring in many medicinal herbs. These have been demonstrated to exert anticancer effects in various cancer cell systems (Yamai et al. 2009). It has been reported that treatment with a combination of anticancer drugs (5-fluorouracil) and triterpenes (betulinic acid, ursolic acid and oleanolic acid) resulted in additive or synergistic suppression of cell growth in vitro and in the reduction of experimental esophageal squamous carcinoma cells cell metastasis by direct suppression of cell growth in vivo (Yamai et al. 2009). Betulinic acid also resulted in suppression of cell growth in vitro and in the prevention of B16F10 melanoma experimental metastasis by suppression of cell growth in vivo (Sawada et al. 2004). In addition, ursolic acid also suppressed expression of MMP-2, as well as declined invasion and migration in these lung and gastric cancer cells (Hussain et al. 2017). Oleanolic acid decreased the expression of the angiogenic vascular endothelial growth factor (VEGF) and decreased the development of melanoma-induced lung metastasis (Žiberna et al. 2017).

In conclusion, we have demonstrated that EAAC inhibits the migration and invasion of carcinoma cancer SK-Hep1 cells. Mechanistically, we show that this effect of EAAC may occur through inactivation of the MAPK signaling pathway, exerting inhibitory effects on FAK and pFAK protein expressions and inhibiting PI3K, Akt, and phospho-Akt levels, thereby decreasing the activities of MMP-2 and MMP-9 leading to inhibition of metastasis of SK-Hep1 cells. These results also demonstrate the inhibition of both MMP-2 and MMP-9 activities process by EAAC may be helpful in developing new chemotherapeutic strategies for SK-Hep1 cell related human hepatoma.

## Abbreviations

EAAC: ethyl acetate fraction of *Actinidia callosa* var. *callosa*
MMP-2/9: metalloproteinase-2/9
TIMP-1/2: metalloproteinase-1/2
MAPK: mitogen-activated protein kinase
PI3K: phosphatidylinositol-3-kinase
AKT (PKB): protein kinase B
FAK: focal adhesion kinase
